# Efficacy of intravenous tranexamic acid administration in medial opening-wedge distal tibial tuberosity osteotomy (MOWDTO) for varus knee osteoarthritis: a randomized control trial

**DOI:** 10.1186/s13018-023-03666-z

**Published:** 2023-03-08

**Authors:** Takuya Iseki, Tomoya Iseki, Ryo Kanto, Shintaro Onishi, Shinichi Yoshiya, Toshiya Tachibana, Hiroshi Nakayama

**Affiliations:** 1 Department of Orthopaedic Surgery, Hyogo Medical University, 1-1 Mukogawa-Cho, Nishinomiya City, Hyogo 663-8501 Japan; 2 Department of Orthopaedic Surgery, Nishinomiya Kaisei Hospital, 1-4 Ohama-Cho, Nishinomiya City, Hyogo 662-0957 Japan

**Keywords:** Tranexamic acid, Opening-wedge distal tibial tuberosity osteotomy (MOWDTO), Randomized controlled study

## Abstract

**Background:**

This randomized controlled study was undertaken to investigate the efficacy of intravenous tranexamic acid (TXA) administration in reducing perioperative blood loss in patients undergoing medial opening-wedge distal tibial tuberosity osteotomy (MOWDTO). It was hypothesized that TXA would reduce perioperative blood loss in MOWDTO.

**Methods:**

A total of 61 knees in 59 patients who underwent MOWDTO during the study period were randomly assigned to either of the groups with intravenous TXA administration (TXA group) or without TXA administration (control group). In the TXA group, patients received 1000 mg of TXA intravenously before skin incision and 6 h after the first dose. The primary outcomes was the volume of perioperative total blood loss which calculated using the blood volume and hemoglobin (Hb) drop. The Hb drop was calculated as the difference between preoperative Hb and postoperative Hb at days 1, 3, and 7.

**Results:**

The perioperative total blood loss was significantly lower in the TXA group (543 ± 219 ml vs. 880 ± 268 ml*, P* < 0.001). The Hb drop was significantly lower at postoperative days 1, 3 and 7 in the TXA group than in the control group (day 1: 1.28 ± 0.68 g/dl vs. 1.91 ± 0.69 g/dl, *P* = 0.001; day 3: 1.54 ± 0.66 g/dl vs. 2.69 ± 1.00 g/dl, *P* < 0.001; day 7: 1.74 ± 0.66 g/dl vs. 2.83 ± 0.91 g/dl, *P* < 0.001).

**Conclusion:**

Intravenous TXA administration in MOWDTO could reduce the perioperative blood loss.

*Trial registration* The study was approved by the institutional review board. (Registered on 26/02/2019 Registration Number 3136).

*Level of Evidence* Level I, randomized controlled trial.

## Introduction

Osteotomy around the knee is a widely employed surgical option for medial compartment knee osteoarthritis exhibiting varus deformity [1, 2]. However, the complications such as delayed wound healing, infection, and hematoma are still problematic in knee osteotomies [3–6]. Excessive postoperative bleeding can be an initiating factor leading to those complications associated with subcutaneous hematoma and delayed wound healing as subcutaneous soft tissue coverage on the osteotomy side is limited [6, 7]. Additionally, some studies have reported that there were some cases that required blood transfusions in medial opening-wedge high tibial osteotomy (MOWHTO) [8–10]. Therefore, perioperative bleeding management would be an essential measure to be undertaken to reduce the incidence of those postoperative problems.

As a countermeasure for postoperative bleeding, tranexamic acid (TXA) administration plays a key role in various surgical fields [11, 12]. In recent years, a number of randomized controlled studies have revealed the effectiveness of TXA in reducing perioperative blood loss in patients undergoing total joint arthroplasty [13, 14], and a consensus has been reached regarding its efficacy in this patient population. By contrast, there have only been two randomized controlled trials dealing with this subject in osteotomies around the knee [10, 15]. Consequently, clinical evidence validating routine TXA administrations in knee osteotomies is still not robust enough.

**Fig. 1:**
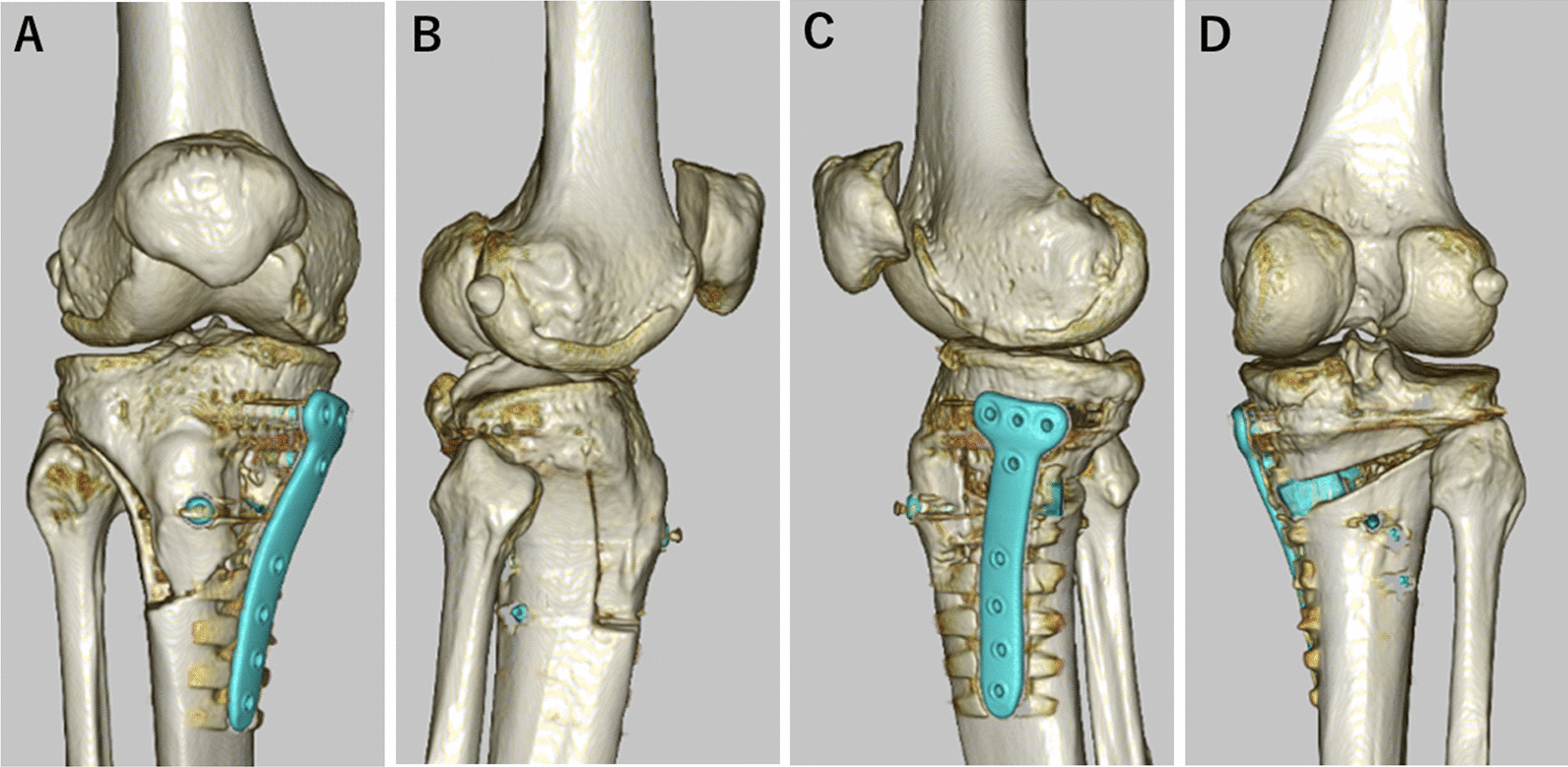
Postoperative three-dimensional computed tomography after medial opening-wedge distal tibial tuberosity osteotomy for right knee. **A** anteroposterior view. **B** lateral view. **C** medial view. **D** posteroanterior view

With the recent trend in joint preservation and biological reconstruction in surgical treatment of the knee, the number of types of osteotomy procedures being performed has been increasing in recent years [16–19]. In our clinical practice, we have adopted opening-wedge distal tibial tuberosity osteotomy (MOWDTO) developed by Akiyama et al*.* [20], which is a modification of HTO, as the primary surgical option for osteotomy with mild to moderate varus knee osteoarthritis (Fig. 1). Several clinical studies have reported clinical advantages of MOWDTO over conventional MOWHTO [21–23]; however, there is concern of increased bleeding from the bone marrow compared to MOWHTO due to the triplane osteotomy technique which requires three osteotomy surface areas. To the best of our knowledge, there has been no randomized controlled trials that investigated the effectiveness of TXA administration in MOWDTO.

The purpose of this study was to investigate the efficacy and safety of TXA administration in MOWDTO by examining the perioperative amount of bleeding and the incidence of postoperative deep vein thrombosis (DVT). The hypothesis of this study was that TXA would reduce perioperative total blood loss without increasing the risk of DVT.

## Materials and methods

### Study design

This prospective, two-arm, parallel group, randomized-controlled, open-label trial with 1:1 treatment allocation was performed in a single university hospital. The study was approved by the institutional review board. (Registered on 26/02/2019, registration number 3136). Before participant enrollment, the trial was registered as a randomized controlled trial with the University Hospital Medical Information Network (Registered on 9/11/2018, registration number UMIN000034842).

Patients were recruited between February 2019 and March 2021 from the patient population of a single university hospital, and eligible patients gave written informed consent. The inclusion criteria of this randomized controlled trial were patients older than 20 years with medial compartment knee osteoarthritis scheduled for MOWDTO. The exclusion criteria were as follows: previous history of DVT or pulmonary embolism (PE); patients who declared an allergy to TXA by the preoperative interview; patients scheduled for MOWDTO combined with other procedures such as implant removal, anterior cruciate ligament (ACL) reconstruction, osteochondral autograft transfer, and autologous chondrocyte implantation.

### Randomization

Each patient participating in the trial was randomly assigned to one of the two groups through simple random allocation. We generated randomized numbers ranging from 0 to 99 with a computer software (Excel 2010; Microsoft, Redmond, Washington, USA). Patients with even numbers were allocated to the group scheduled to receive intravenous TXA, and those with odd numbers were allocated to receive no TXA administration.

### Interventions

The study treatments were intravenous TXA administration (TXA group) or no administration of TXA (control group). In the TXA group, patients received 1000 mg of TXA diluted by 100 ml of saline intravenously (Transamin; Daiichi Sankyo, Tokyo, Japan) before skin incision and 6 h after the first dose. The patients allocated in the control group did not receive TXA. Other perioperative interventions, such as surgical technique, rehabilitation regimen, and perioperative medications were the same for all patients.

### Surgical procedure

**Fig. 2:**
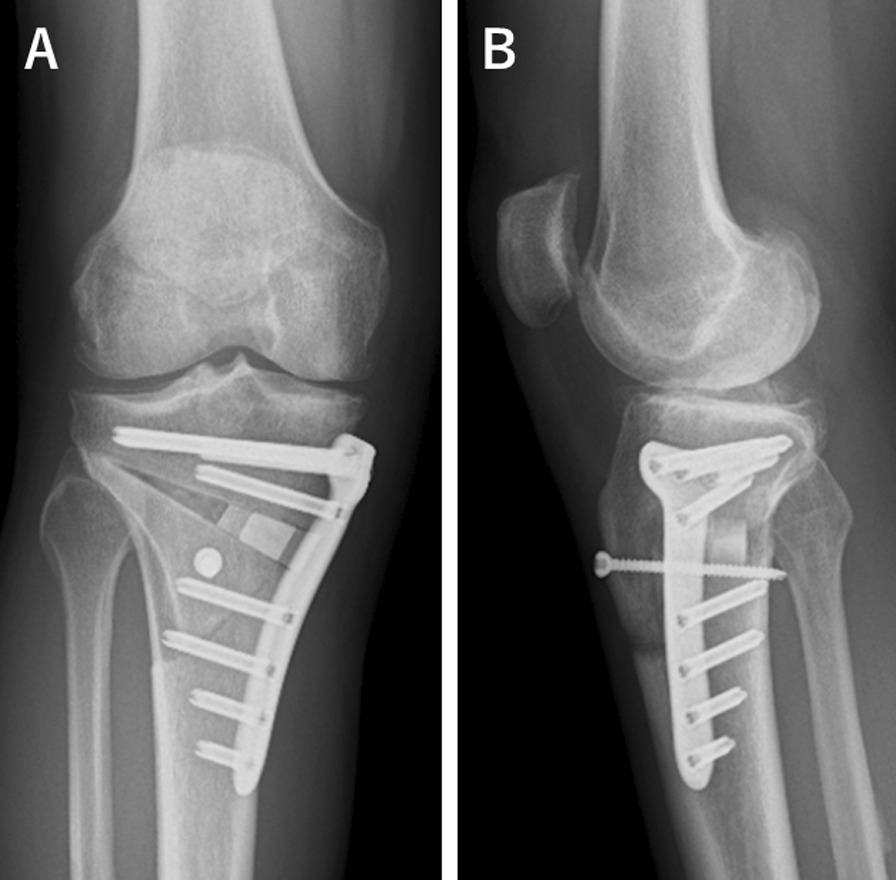
Postoperative radiograph of the right knee. **A** anteroposterior view. **B** lateral view

All surgical procedures were performed in a standardized manner under general anesthesia and performed by one of the four surgeons. No pneumatic tourniquet was used during the study period. The MOWDTO procedure performed in this study followed the triplane osteotomy technique described by Akiyama T [20]. First, transverse osteotomy was made along the guide wire inserted toward the hinge point, which was located at the upper level of the proximal tibiofibular joint and 5 mm medial from the lateral cortical margin. The osteotomy was initiated from 35 to 40 mm distal to the medial tibial joint surface. Subsequently, an arc osteotomy centered at the hinge point was performed with a radius of approximately 50 mm. To connect the arc osteotomy with the first transverse osteotomy, a descending osteotomy was made on the coronal plane starting at 15 mm posterior to the tibial tubercle and advancing distally to the level of the arc osteotomy (Fig. 1). After the triplane osteotomy was completed, the medial wedge was gradually opened while monitoring the limb alignment on fluoroscopy by checking the position of the alignment rod at the joint level. Finally, the osteotomy gap was filled with β-tricalcium phosphate (OLYMPUS, Tokyo, Japan) and fixed using a locking plate (Tris Medial HTO Plate System; OLYMPUS, Tokyo, Japan) (Fig. 2). The suction drain was placed in the subcutaneous layer.

### Postoperative care

After surgery, elastic compression stockings and a mechanical compression device were applied to the lower limbs on both sides. For chemoprophylaxis for postoperative thromboembolism, 15 mg per day of edoxaban, an Xa-inhibitor, was orally administered until one week after surgery. At one week, ultrasonographic examination with pulsed Doppler, color Doppler (Toshiba Medical Systems, Japan) was performed for screening of DVT.

Postoperative drainage volume was recorded separately on the first day and second day, and the suction drain was removed on the second day. From the day after surgery, patients were given oral 60 mg of loxoprofen three times a day and 75 mg of pregabalin once a day. For patients with severe pain, additional prescription of tramadol hydrochloride acetaminophen was allowed as needed.

The postoperative rehabilitation regimens were the same for both groups, and range of motion exercise was initiated at postoperative day 1. Weight bearing of lower limbs on the surgical side was allowed at two weeks. We evaluated hemoglobin (Hb) at postoperative day 1, 3 and 7. Allogeneic blood transfusion was indicated for patients with Hb < 7.0 g/dl or those with Hb < 10.0 g/dl if they had symptoms related to anemia.

### Outcome measures

#### Primary outcome

The primary outcome was the volume of perioperative total blood loss, which was calculated as follows.

First, the blood volume of the patient was calculated in liters using the following formula according to Nadler et al*.* [24].

$${\text{Blood}}\;{\text{volume }} = \, \left( {{\text{k1 }} \times {\text{ height }}\left[ {\text{m}} \right]^{{3}} } \right) \, + \, \left( {{\text{k2 }} \times {\text{ body}}\;{\text{weight }}\left[ {{\text{kg}}} \right]} \right) \, + {\text{ k3}}$$

k1 = 0.3669 for male patients and 0.3561 for female patients.k2 = 0.03219 for male patients and 0.03308 for female patients.k3 = 0.6041 for male patients and 0.1833 for female patients.

Second, Hb loss was calculated according to the following formula.

$$\begin{aligned} {\text{Hb}}\;{\text{loss}} = {\text{ blood }}\;{\text{volume }} \times & {\text{ Hb}}\;{\text{ drop }}\;{\text{corresponding }}\;{\text{to }}\;{\text{the }}\;{\text{difference}}\;{\text{ between }} \\ {\text{the}}\;{\text{ preoperative }}\;{\text{Hb }}\;{\text{and }}\;{\text{the}}\;{\text{ minimum }}\;{\text{postoperative }}\;{\text{Hb}} \times \, 0.00{1} \\ \end{aligned}$$

Finally, the total blood loss was calculated as follows:

$${\text{Total}}\;{\text{ blood}}\;{\text{ loss }} = { 1}000 \, \times {\text{ Hb}}\;{\text{ loss}}/{\text{ preoperative}}\;{\text{ Hb}}$$

In addition, Hb drop at postoperative days 1, 3 and 7 and the amount of postoperative drainage volume were measured and compared between two groups.

#### Secondary outcomes

The secondary outcomes were the overall incidence of DVT, rate of blood transfusion, the incidence of wound complications and superficial soft tissue infections, leg circumference and the visual analog scale (VAS) score. The circumference of the leg was measured before surgery and at postoperative day 7 by physiotherapists at the maximum circumference of the lower leg. The VAS score at rest was measured periodically from the following day to five days after surgery at 6:00 AM, 12:00 PM, and 8:00 PM. The VAS score during activity was defined as the strongest pain experienced during physical therapy.

#### Statistical analyses and sample size

Statistical analyses were performed using the R software (The R Foundation for Statistical Computing). The comparisons between the two groups were performed using the Student’s *t* test for continuous variables and the Chi-square test for categorical variables. All tests were two-sided, and *P* < 0.05 was considered statistically significant. In previous reports, the mean difference in total blood loss between the TXA group and control group varied from 90 to 260 ml [25, 26]. Based upon these reports, we set the mean difference between two groups for the detection of sample size to 170 ml and calculated that 29 patients per group would be required for this trial.

## Results

### Participants and patient characteristics

**Fig. 3:**
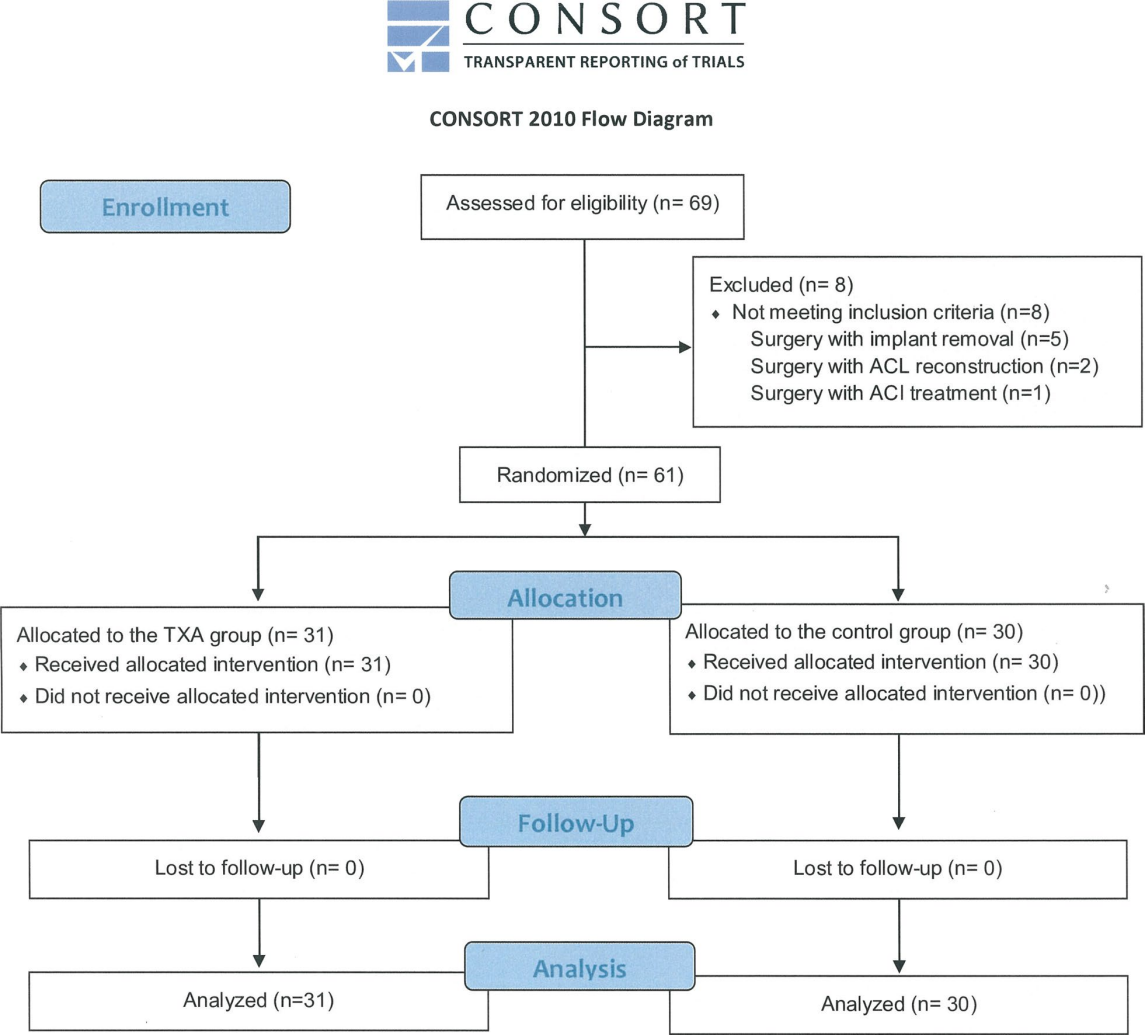
Diagram showing the flow of patients through each stage of the trial

A total of 69 knees in 66 patients underwent MOWDTO during this clinical trial and eight knees were excluded (Fig. 3). Finally, 61 knees in 59 patients meeting the inclusion/exclusion criteria were randomly assigned with 31 knees allocated to the TXA group and 30 knees to the control group. The demographic characteristics of the patients in the two groups are presented in Table 1, and no statistically significant differences were noted between the groups.

**Table 1:** Patient demographics and baseline clinical characteristics

	TXA group (*n* = 31 knees)	Control group (*n* = 30 knees)	*P-*value
Age (years)	57.4 ± 8.7	58.0 ± 8.6	0.805
Sex (female/male)	15/16	16/14	0.800
Height (cm)	161 ± 8.8	164 ± 6.5	0.139
Weight (kg)	67.7 ± 17.3	70.6 ± 16.2	0.499
BMI (kg/m^2^)	25.8 ± 4.9	26.1 ± 5.0	0.855
Preoperative Hb (g/dl)	14.2 ± 1.3	14.2 ± 1.3	0.884
Preoperative Ht (%)	41.7 ± 3.7	42.0 ± 3.0	0.743
Preoperative D-dimer (mg/dl)	0.5 ± 0.4	0.4 ± 0.1	0.294
Preoperative lower leg circumference	37.4 ± 3.6	36.3 ± 3.3	0.354
Preoperative VAS at rest	11.0 ± 16.6	16.1 ± 24.0	0.378
Duration of surgery (min) Medial opening gap width (mm)	84.1 ± 16.8 8.9 ± 3.4	83.7 ± 23.2 8.4 ± 2.5	0.934 0.537

Results are expressed as means ± standard deviation

*TXA* tranexamic acid, *BMI* body mass index, *Hb* hemoglobin, *VAS* visual analogue scale

### Primary outcome

The volume of perioperative total blood loss was 543 ± 219 ml and 880 ± 268 ml in the TXA group and in the control group, respectively, with a statistically significant difference between the two groups (*P* < 0.001). The Hb drop at postoperative days 1, 3 and 7 was significantly lower in the TXA group than in the control group (day 1: 1.28 ± 0.68 g/dl vs. 1.91 ± 0.69 g/dl, *P* = 0.001; day 3: 1.54 ± 0.66 g/dl vs. 2.69 ± 1.00 g/dl, *P* < 0.001; day 7: 1.74 ± 0.66 g/dl vs. 2.83 ± 0.91 g/dl, *P* < 0.001). The postoperative drainage volume on the first day was significantly lower in the TXA group (*P* < 0.001) (Table 2).

**Table 2:** The perioperative total blood loss, hemoglobin loss, and drainage volume

	TXA group (*n* = 31 knees)	Control group (*n* = 30 knees)	*P-*value
Total blood loss (ml)	543 ± 219	880 ± 268	< 0.001*
*Hb drop (g/dl)*			
Postoperative day 1 Postoperative day 3 Postoperative day 7	1.28 ± 0.68 1.54 ± 0.66 1.74 ± 0.66	1.91 ± 0.69 2.69 ± 1.00 2.83 ± 0.91	0.001* < 0.001* < 0.001*
*Drainage volume (ml)*			
0–24 h after surgery 24–48 h after surgery Total volume	179 ± 101 122 ± 84 300 ± 141	293 ± 149 96 ± 73 386 ± 189	< 0.001* 0.217 0.051

Results are expressed as means ± standard deviation

*TXA* tranexamic acid, *Hb* hemoglobin

*Significant difference between the groups

### Secondary outcomes

**Fig. 4:**
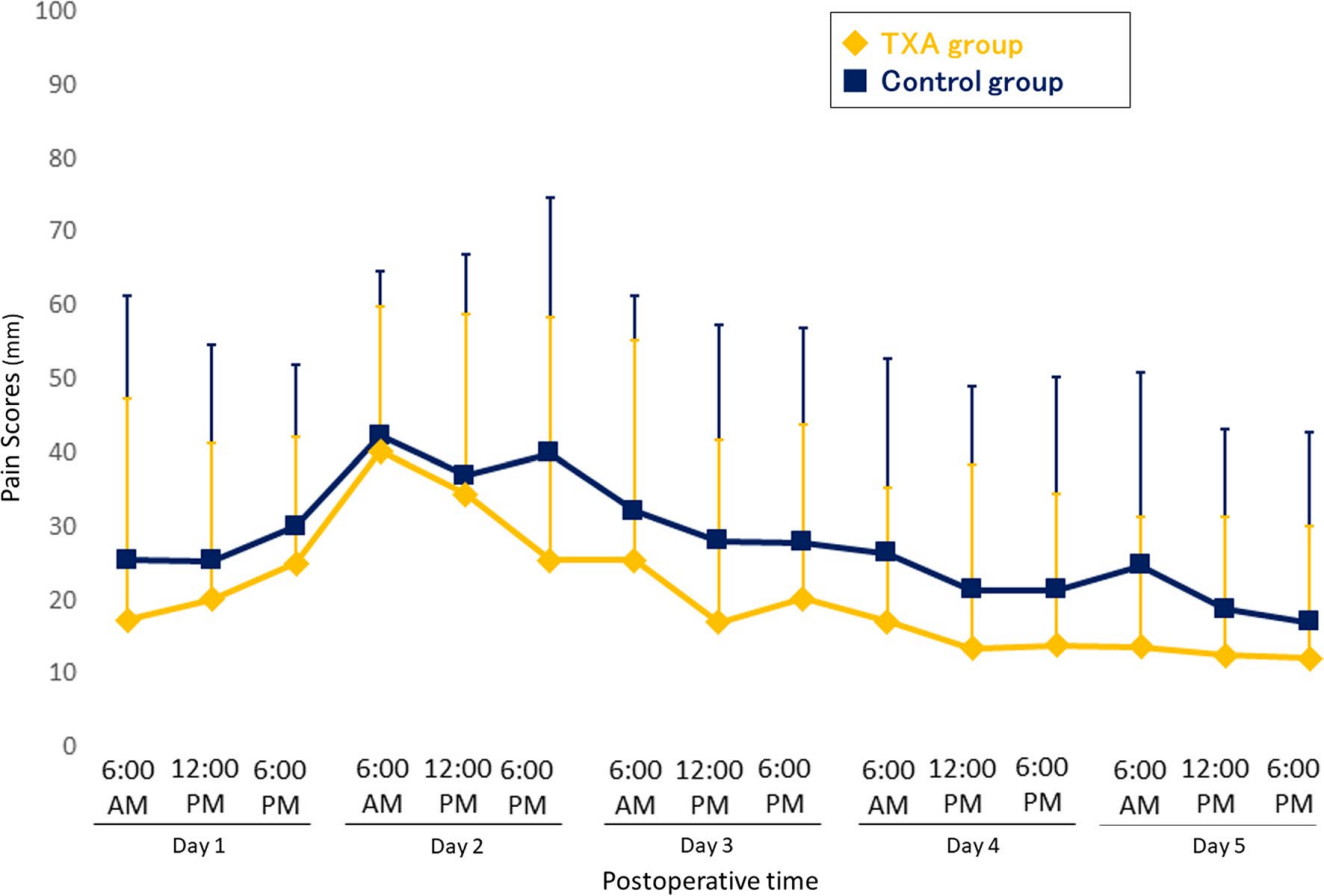
Visual analog scale scores for pain at rest after surgery

**Fig. 5:**
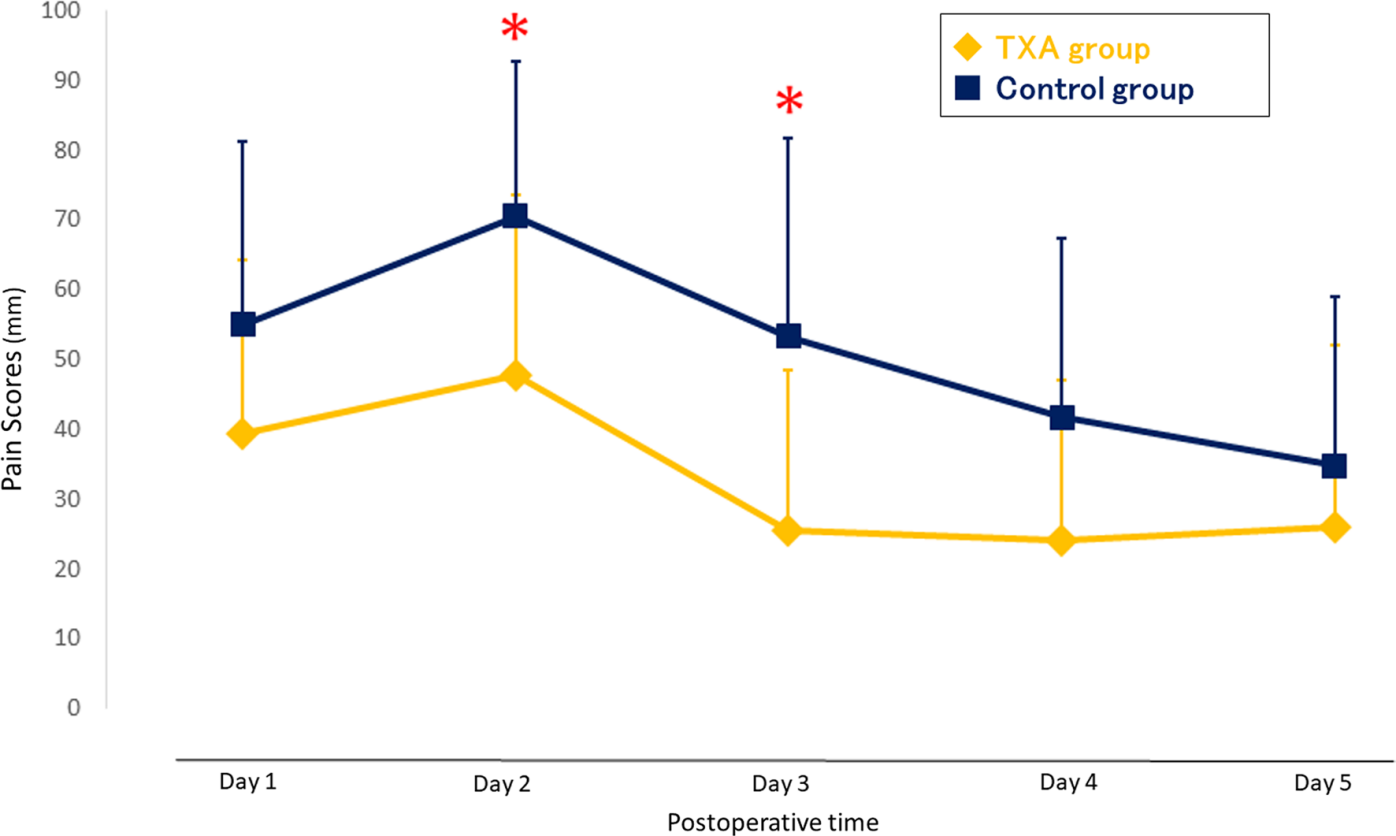
Visual analog scale scores for pain during activity after surgery

The ultrasonographic examination at postoperative day 7 revealed DVT in the lower leg in two patients in the TXA group and two patients in the control group (*P* > 0.999). Neither symptomatic DVT nor pulmonary embolism were encountered in both groups. No patient needed blood transfusions, and there were no wound complications or hematoma requiring additional treatment after surgery. There were no significant differences in lower leg circumference between the TXA group and the control group at postoperative day 7 **(**37.9 ± 4.3 cm versus 37.5 ± 3.8 cm, *P* = 0.723**)**. The VAS score at rest and during activity is shown in Figs. 4 and 5. The mean VAS scores at rest showed no significant difference between two groups (Fig. 4). The mean VAS scores during activity in the TXA group were significantly lower on postoperative day 2 and day 3 (day 2: 47.7 ± 26.2 vs. 70.5 ± 22.3, *P* = 0.017; day 3: 25.6 ± 23.2 vs. 53.3 ± 28.5, *P* = 0.006) than that in the control group as indicated by the red asterisk in Fig. 5. Two patients in both groups were prescribed additional tramadol hydrochloride acetaminophen on postoperative day 2 for severe pain.

## Discussion

The most important finding of this study was that the intravenous administration of TXA reduced perioperative total blood loss in MOWDTO without increasing the incidence of DVT.

This is the first randomized controlled study to examine the effectiveness of intravenous TXA administration in MOWDTO, which was developed as a modification of MOWHTO.

Osteotomies around the knee are widely accepted surgical options for osteoarthritic knees and there are several modified procedures to cope with potential shortcomings of MOWHTO. However, some authors have reported that approximately 5 to 7% of patients who underwent MOWHTO experienced complications associated with wound problems [3, 4] and some patients needed blood transfusions [9, 14]. Therefore, a proper perioperative bleeding management and reduction of blood loss would be essential to reduce these risks and enhance patient satisfaction after knee osteotomy. Especially in performing MOWDTO, there is an increased risk of bleeding from the intramedullary caused by the triplane osteotomy technique, which require a wider osteotomy surface area than MOWHTO, and implementation of strategies should be taken into consideration. The efficacy of TXA administration in reducing the amount of perioperative blood loss has been well established in various orthopedic procedures such as joint arthroplasty and spine surgery [13, 14]. There have been several studies examining the efficacy and safety of TXA administration in knee osteotomies [10, 15, 27]. Kim et al. demonstrated that the administration of TXA reduced total estimated blood loss and total amount of drainage after MOWHTO [9]. Palanisamy et al. also reported that the administration of TXA contributed to the reduction of total blood loss, Hb drop and drain output after MOWHTO [26]. However, pooled evidence in literatures is still not enough, moreover, as addressed in a meta-analysis paper by Ma et al. [28], the majority of those studies represent retrospective comparative analyses and a well-designed randomized controlled trial is warranted. In the present study, randomization was performed to demonstrate if the intravenous TXA administration was effective. Consequently, the obtained results validated that TXA could decrease perioperative bleeding in MOWDTO. Regarding blood transfusions, in this study, there were no patients who needed blood transfusions in either groups. However, several authors reported that some patients who did not receive TXA required blood transfusions in MOWHTO [9, 10].

As for potential risks of the use of TXA, previous studies in patients undergoing total joint arthroplasty have shown that TXA administration is not associated with increased incidence of DVT and PE [29, 30]. Regarding the MOWHTO, Kim et al. investigated postoperative DVT by computed tomography venography of the lower limbs extremities and reported that there were no cases of postoperative DVT in patients who received TXA intravenously [9]. Corresponding to those studies, the present study showed no significant difference in the incidence of DVT as screened by ultrasonography between two groups. Therefore, it seems that the administration of TXA can be safely used in osteotomies around the knee in terms of the development of DVT.

Regarding the effectiveness of TXA administrations in decreasing postoperative pain, several reports have concluded that it could relieve postoperative pain [31–33]. Wang et al. reported that TXA had an anti-inflammatory effect due to its inhibition of plasmin, and that administration of TXA reduced inflammatory markers as manifested by the suppression of interleukin-6, the erythrocyte sediment rate and C-reactive protein levels [34]. Moreover, they also reported that postoperative maintenance administration of TXA may provide additional anti-inflammatory benefits and that a positive relationship between these inflammatory markers and postoperative pain. In the present study, an additional dose administered 6 h after the first dose may have contributed to a reduction in the mean pain VAS score during activity on postoperative days 2 and 3.

There are several limitations in this study. First, the surgical team and anesthesiologists were not blinded. On the other hand, the surgical approach was consistent in both groups, and the total blood loss as a primary outcome could not have been biased by the observer. Second, the control group did not receive placebo. Finally, in the present study, the obtained data are limited to only short-term outcomes and the effects of TXA administration on the clinical outcome or patient satisfaction were not subjected to analysis.

## Conclusion

Intravenous TXA administration in MOWDTO reduced the perioperative blood loss without increasing the risk of DVT. These study results suggest that intravenous administration of TXA might be an effective and safe measure for control of perioperative bleeding in this modified osteotomy procedure.

## Data Availability

The datasets used and/or analyzed during the current study are not publicly available due to their containing information that could compromise the privacy of research participants. However, the data are available from the corresponding author on reasonable request.

## Abbreviations

TXA: Tranexamic acid
MOWDTO: Medial opening-wedge distal tibial tuberosity osteotomy
